# Repolarization Heterogeneity: Beyond the QT Interval

**DOI:** 10.1161/JAHA.116.003607

**Published:** 2016-04-29

**Authors:** Stuart B. Prenner, Sanjiv J. Shah, Jeffrey J. Goldberger, Andrew J. Sauer

**Affiliations:** ^1^ Division of CardiologyBluhm Cardiovascular InstituteNorthwestern Memorial HospitalNorthwestern University Feinberg School of MedicineChicagoIL; ^2^ Division of CardiologyMiller School of MedicineUniversity of MiamiFL; ^3^ Division of CardiologyUniversity of Kansas School of MedicineKansas CityKS

**Keywords:** arrhythmia, electrocardiography, electrophysiology, heart failure, mechanics, Electrophysiology, Electrocardiology (ECG), Heart Failure

Research over the past 2 decades has suggested that significant differences exist in the action potentials of endocardial, epicardial, and mid‐myocardial (M) cells that comprise the ventricular myocardium. Relative differences in the time course of repolarization of these 3 cell types, referred to as transmural dispersion of repolarization (TDR), is largely responsible for the inscription of the T wave on the electrocardiogram (ECG). Both the morphology and duration of the T wave appear to represent underlying heterogeneity in repolarization, a process initially linked to increased risk of arrhythmia and sudden death in patients with congenital long QT‐syndrome (LQTS). It is increasingly apparent that repolarization heterogeneity is present in a variety of cardiovascular diseases and syndromes and is even predictive of sudden death in the general population. Furthermore, repolarization heterogeneity has also been associated with abnormalities in myocardial mechanics, a finding that may have direct implications for understanding the pathophysiology and treatment of heart failure. This review will focus on summarizing current understanding of repolarization heterogeneity, with particular focus on clinical implications.

## Physiology of Myocardial Repolarization Heterogeneity

It has been nearly 3 decades since the description of the M cell reframed understanding that regional differences exist in the electrical properties of the ventricular myocardium. Canine models developed in the early 1990s for studying ventricular action potentials first identified electrophysiologically distinct mid‐myocardial cells, termed M cells, which were found to exhibit unique repolarization properties compared to the cells contained in the endocardium and epicardium.^1^, ^2^ M cells are similar to epicardial and endocardial cells histologically, but electrophysiologically and pharmacologically appear to be a hybrid between Purkinje and ventricular cells. The hallmark of the M cell is the characteristic of its action potential (AP) to prolong disproportionately relative to the action potential of other ventricular myocardial cells in response to heart rate slowing and action potential duration (APD)‐prolonging agents.^1^

The ionic basis for these M‐cell features include the presence of a smaller slowly activating delayed rectifier potassium (K^+^) current (I_Ks_), a larger late sodium (Na^+^) current, and a larger Na^+^‐calcium (Ca^2+^) exchange current.^3^, ^4^ Accordingly, there are prominent differences between M cells and the surrounding myocardial cells in the response to various drugs. Alpha‐adrenergic agonists, such as phenylephrine, produce a prolongation of Purkinje APD, whereas they abbreviate the M‐cell APD.^5^ Rapidly activating delayed rectifier K^+^ current (I_Kr_) blockers, including d‐sotalol and erythromycin, produce a much greater prolongation of APD in M cells than cells in the epicardium or endocardium. Other differences include the mechanisms of development of early afterdepolarizations (EADs). EADs induced in the M cell are more sensitive to changes in intracellular Ca^2+^ levels, whereas EADs elicited in Purkinje cells are not.^6^ These differences at the cellular level allow for development of larger regional repolarization variations that were finally demonstrated in vitro through development of an arterially perfused myocardial wedge preparation.

**Figure 1:**
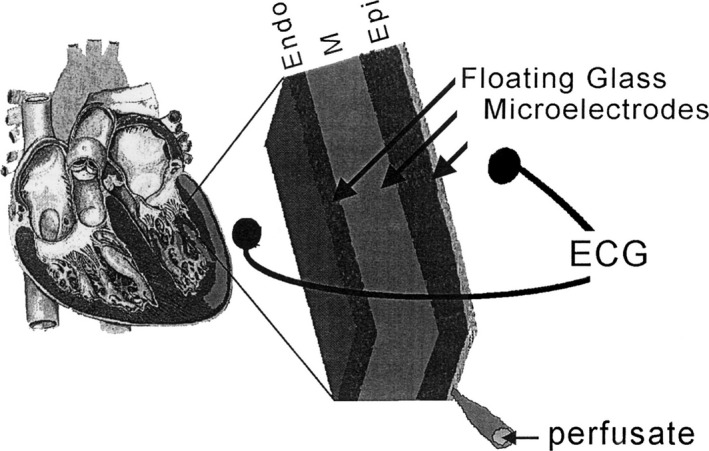
Arterially perfused left ventricular wedge model of canine myocardium. Schematic diagram of the arterially perfused canine LV wedge preparation. The wedge is perfused by a small native branch of the left descending coronary artery and stimulated from the endocardial surface. Transmembrane action potentials are recorded simultaneously from epicardial (Epi), M region (M), and endocardial (Endo) sites using three floating microelectrodes. A transmural ECG is recorded along the same transmural axis across the bath, registering the entire field of the wedge. Reproduced with permission from Yan et al.^9^ Promotional and commercial use of the material in print, digital, or mobile device format is prohibited without the permission from the publisher Wolters Kluwer Health. ECG indicates electrocardiogram; LV, left ventricular.

In vivo models in dogs and rabbits have the disadvantage of requiring anesthesia, which can directly affect myocardial conduction and repolarization.^7^, ^8^ Anesthetics, including sodium pentobarbital, were found to be protective of torsades de pointes (TdP) in dogs, whereas halothane offered no protection.^7^, ^8^ Development of an arterially perfused left ventricular (LV) myocardial wedge preparation allowed for examination of the interaction between these cell types under physiological conditions, without the confounding effects of anesthesia (Figure 1).^9^ The qualitative differences between the 3 ventricular cell types previously described in isolated tissues and cells are present in the intact canine LV wall preparations.^9^ TDR is the result of intrinsic differences in APD of cells spanning the ventricular wall. In the absence of electrolyte or pharmacological provocation, myocytes isolated from the M region display APDs as much as several 100 ms longer than those recorded from the endocardium or epicardium. As a result, transmural dispersion represents the net differences in repolarization times between these distinct action potentials. Accordingly, drugs such as sotalol and erythromycin, which preferentially prolong M‐cell APD, will increase dispersion of repolarization dramatically.^10^

**Figure 2:**
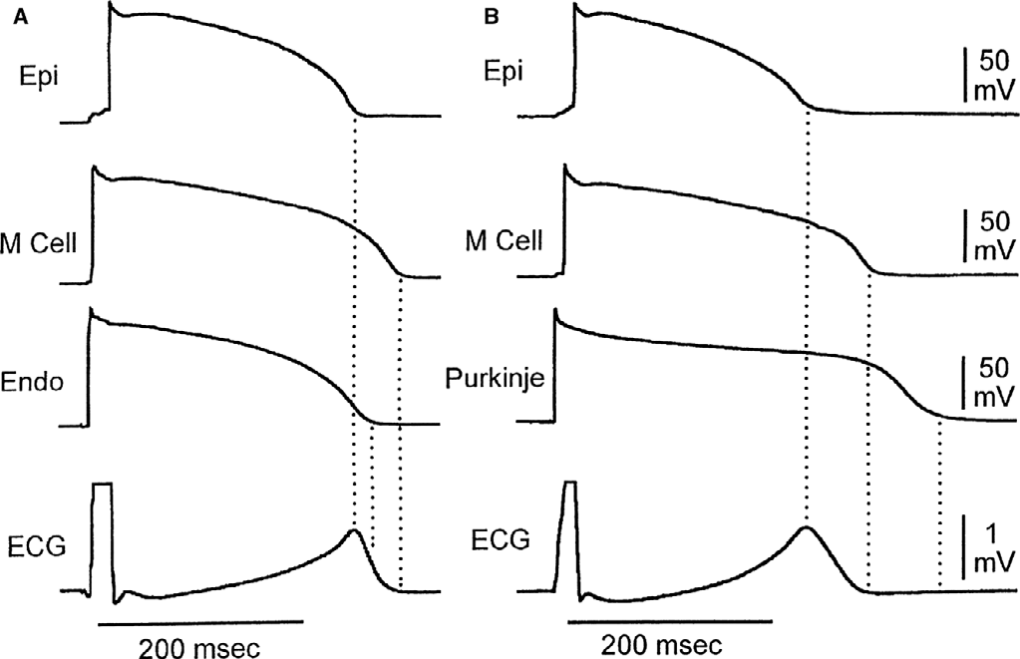
Cellular basis for normal T‐wave inscription. Shown here is the temporal relationship between transmembrane action potentials recorded from epicardial, M region, and endocardial (A) or subendocardial Purkinje fiber regions (B). Note that M cell repolarization is aligned with end of the T wave, whereas repolarization of the epicardial cells is coincident with the peak of the T wave. ECG indicates electrocardiogram; Endo, endocardial; Epi, epicardial; M Cell, Masonic myocardial Moe cell. Reproduced with permission from Yan et al.^10^ Promotional and commercial use of the material in print, digital, or mobile device format is prohibited without the permission from the publisher Wolters Kluwer Health.

**Figure 3:**
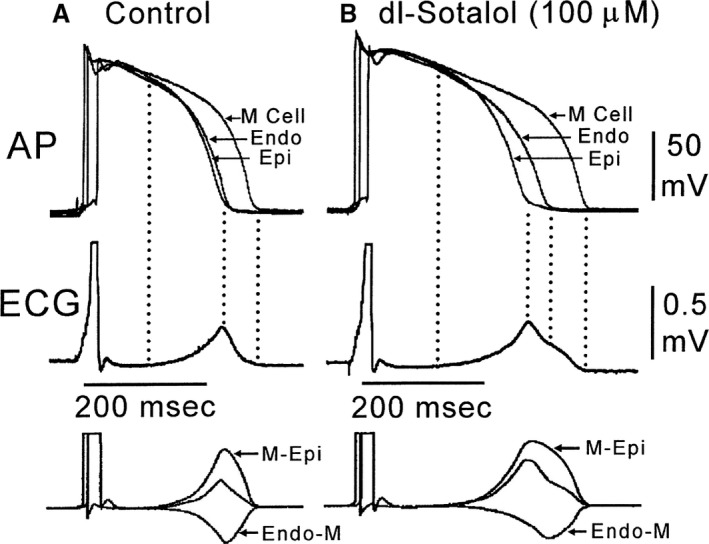
Transmural dispersion of repolarization. Shown here are the baseline (A) and sotalol‐induced changes (B) in APD of each layer of the canine left ventricular arterially perfused wedge. Note the disproportionately prolonged M‐cell action potential and its corresponding contribution to the prolongation of the time from the peak to the end of the T wave (T_peak−end_). Note as well the bifurcated or notched T‐wave morphology. The bottom of the figure shows the calculated voltage differences between epicardial and M‐cell APs (M‐Epi) and between the M‐cell and endocardial responses (Endo‐M) (bottom). AP indicates action potential; APD, action potential duration; ECG, electrocardiogram; Endo, endocardial; Epi, epicardial; M Cell, Masonic myocardial Moe cell. Reproduced with permission from Yan et al.^10^ Promotional and commercial use of the material in print, digital, or mobile device format is prohibited without the permission from the publisher Wolters Kluwer Health.

Subsequent studies found that the currents flowing adjacent to M cells are, in large part, responsible for the morphology of the ECG T wave (Figure 2).^10^ The interplay between these opposing currents determines the height of the T wave as well as the degree to which either the ascending or descending limb of the T wave is interrupted, causing a bifurcated or notched appearance (Figure 3).^10^ Under basal conditions, the epicardial cells are always the earliest to repolarize and the M cells are the last. Full repolarization of the M‐cell region marks the end of the T wave.^10^ The time interval between the peak and end of the T wave, referred to as T_peak_−T_end_ (TpTe), therefore represents the surface ECG manifestation of dispersion of repolarization across the ventricular wall, hereafter referred to as repolarization heterogeneity.

## Electrocardiographic Assessment of Repolarization Heterogeneity

The most often used ECG markers for measuring repolarization heterogeneity include TpTe and QT dispersion (QTD). TpTe is calculated by measuring the interval from the peak of the T wave to its offset. The offset of the T wave is frequently defined as the intersection of the tangent to the steepest portion of the terminal portion of the T wave and the isoelectric line.^11^ Most typically, lead V5 is used because previous studies have suggested that precordial leads best reflect repolarization heterogeneity across the ventricular wall, in contrast to limb leads, which reflect apical‐basal or interventricular spatial heterogeneity.^12^ A large increase in TDR is likely to be arrhythmogenic because the dispersion of repolarization and refractoriness occurs over a very short distance (the width of the ventricular wall), creating a steep repolarization gradient. Difficulties can arise in measuring the exact duration of TpTe, particularly with low T‐wave amplitude, or when T waves are notched or biphasic.^9^, ^13^

Whereas the canine wedge model suggested that TpTe was an accurate measure of “transmural” dispersion, studies of in vivo animal models have suggested that the generation of the T wave may be more complex. Work done by Xia et al. revealed that in open‐chest pig models, the peak of the T wave often occurred 30 to 40 ms before the full repolarization of the epicardium. They concluded that the peak of the T wave likely represented a summation of repolarization gradients both transmurally and apicobasally.^14^ Opthof et al. also showed that TpTe intervals did not correlate with TDR, but did correlate with global dispersion of repolarization in the whole heart.^15^ Validation of TpTe as a direct measure of TDR is still debated, but most studies agree that TpTe provides at least some measure of spatial dispersion or repolarization heterogeneity.

Another potential measure of TDR is QTD. QTD is calculated as the difference between the maximum and minimum QT intervals measured on all 12 leads of the ECG. Reported values of QTD vary widely, with studies showing normal values between 10 and 71 ms.^16^ Work in rabbits revealed that QTD showed significant correlation with dispersion of monophasic AP.^17^ Higham et al. found a high positive correlation between the monophasic action potentials and ECG dispersion indices.^18^ There has been argument, however, that the main cause of QTD may, in fact, be the unreliable localization of the T‐wave offset in patients with abnormal T waves.^19^ This was underscored in a study by Malik et al., which revealed that although QTD differed between healthy controls and patients with dilated cardiomyopathy and ischemic heart disease, QTD was not correlated with T‐wave residuum (TWR).^20^ TWR and several other ECG metrics for quantifying TDR have been proposed and are summarized inTable. This suggests that structural heart disease may be associated with more‐abnormal T‐wave loops, increased difficulty in measuring T‐wave offset, and hence increased QTD indices. However, QTD per say may not represent underlying heterogeneity in repolarization and does not itself confer increased cardiovascular risk.

**Table 1:** Electrocardiographic Measures of Repolarization Heterogeneity

ECG Measure	Definition
Principal component analysis of the T wave	Ratio of the second to first eigenvalues of the spatial T‐wave vector generated from the 12‐lead digital ECG
QRS‐T angle	Adding the mean vector representing all of the electrical forces produced by depolarization and repolarization. This is accomplished by forming a parallelogram using the QRS vector and the T‐wave vector as its sides; the diagonal of the figure is the spatial ventricular gradient.
QT dispersion	Difference in ms between maximal and minimal QTc intervals from between 3 and 6 leads in a simultaneous 12‐lead ECG
Simplified QRS‐T angle	Absolute difference between the QRS and T‐wave axes on the 12‐lead ECG
T peak T end	Time in ms between the peak of the T wave to the end of the T wave, as defined by the intersection of the tangent to the down slope of the T wave and the isoelectric line. Typically measured in V5
T‐wave area (total and late)	Area between the curve and baseline from J point to T end and T peak to T end, respectively
T‐wave residuum	Absolute value of the sum of the squares of the fourth to eighth eigenvalues of the reconstructed T wave after singular value decomposition
T‐wave loop dispersion	Dissimilarities between the T‐wave shapes in individual leads, based on reconstruction vectors of individual ECG leads
Total cosine R‐to‐T	Calculating cosine values between the 3‐dimensional R‐ and T‐wave loop vectors

ECG indicates electrocardiogram.

## Mechanisms of Repolarization Heterogeneity in Congenital and Acquired LQTS

Much of the current understanding of the arrhythmic risk associated with repolarization heterogeneity comes from work in patients with LQTS, both congenital and acquired. In a landmark work, Moretti et al. linked congenital and physiology models of LQTS. The investigators induced pluripotent stem cells from family members affected by LQTS‐1 and directed differentiation into cardiomyocytes. Differentiated cardiomyocytes exhibited electrophysiologic features of the LQTS, including prolonged APD. This was associated with a 70% to 80% reduction in I_Ks_ current and altered channel activation and deactivation properties. This work linked congenital and physiological models of LQTS and helped elucidate underlying molecular mechanisms of arrhythmogenicity.^21^

Congenital LQTS is associated with abnormal T‐wave morphologies on ECG that appear to be specific to the different channel mutations and have even been suggested as a screening tool.^22^ Work with the canine wedge‐preparation model revealed underlying mechanisms for increased arrhythmogenicity of abnormal “LQTS‐type” T waves. Combined I_Kr_ and I_Ks_ blockade simulating LQTS led to development of complex T waves with a late “bump sign.”^13^ When an I_KS_ blocker was used to prolong the QT interval in an LQTS‐1 model, this was not associated with widening of the T wave, increased TDR, or inducible TdP. However, addition of isoproterenol abbreviated the APD of epicardial and endocardial cells, but not that of the M cell, resulting in widening of the T wave and increase in TDR. Only after this exposure to isoproterenol was the myocardium vulnerable to TdP, suggesting that heterogeneously increased APD across the ventricular wall mediates vulnerability to TdP.^23^ I_Kr_ block with sotalol, simulating an LQTS‐2 model resulted in the development of broad‐ and low‐amplitude bifurcated T waves, associated with increased TDR and TpTe in a rate‐dependent manner.^24^ Clinically, TDR is increased in LQT2 more than LQT1, but LQTS‐1 patients show a heart‐rate–dependent increase in TDR.^25^

When observed in other populations without congenital LQTS, the LQTS‐like T‐wave abnormalities described previously seem to similarly represent increase arrhythmic risk. In patients with bradycardia, LQTS‐2‐like T waves were more frequent in those who developed TdP. Furthermore, increased TpTe was highly associated with development of TdP and performed better as a predictor of TdP than either QTc or QT intervals.^26^ Findings were similar in patients with drug‐induced QT‐prolongation, in whom TpTe did not correlate with changes in QTc, suggesting that the arrhythmic risk is not mediated simply by prolonged QTc.^12^ In patients initiated on sotalol, LQTS‐2‐like T‐wave changes developed, which were independent of changes in QTc.^27^ And, when QT does prolong, dispersion of the QT interval, rather than QT prolongation itself, seems to contribute most to arrhythmogenicity.^28^ Thus, work in both congenital and acquired LQTS populations suggested that other ECG markers besides QT interval, including abnormal T‐wave morphologies and increased TpTe are associated with abnormalities in repolarization heterogeneity, and even increased arrhythmic risk.

## Repolarization Heterogeneity as a Risk Factor for Mortality in the General Population

Repolarization heterogeneity is associated with adverse outcomes, including mortality, in the general population. An analysis of 5812 healthy individuals over the age of 55 years in the Rotterdam Study showed that during a 4‐year follow‐up period, those in the highest tertile of QTc dispersion (QTcD) had a nearly 2‐fold increased risk of cardiac death, increased rates of sudden cardiac death (SCD), and overall 40% increased mortality, compared to the lowest tertile.^29^ TpTe was independently associated with SCD in the Oregon Sudden Unexplained Death Study, and a 1‐SD increase in TpTe increased odds of SCD by 3.5‐fold.^30^ As part of the Finnish Health Study, Porthan et al. examined several T‐wave morphology parameters, including principal component analysis (PCA) ratio, T‐wave morphology dispersion, total cosine R‐to‐T (TCRT), and TWR. PCA ratio and T‐wave morphology dispersion were independent predictors of all‐cause and cardiovascular mortality in males. In females, TCRT and TWR were independent predictors of cardiovascular mortality, and TWR was also an independent predictor of all‐cause mortality.^31^

T‐wave markers were also studied in a population of Native Americans as part of the Strong Health Study, in which 1729 Native Americans were followed for a mean of 4.8 years. Increased PCA ratio and TWR were significant predictors of cardiovascular mortality, and TWR was an independent predictor of all‐cause mortality.^32^ The investigators also demonstrated that QTD was a predictor of cardiovascular mortality in females.^33^ Finally, Kardy et al. showed that in 6134 participants in the Rotterdam Study community‐based cohort, increased QRS‐T angle (defined inTable) was independently associated with increase hazard of cardiac death, sudden death, and total mortality.^34^

## Repolarization Heterogeneity in Specific Populations

Repolarization heterogeneity has also been linked to adverse outcomes in numerous cardiovascular disease populations. In patients presenting with acute coronary syndromes (ACSs), repolarization heterogeneity has been associated with increased risk of fatal arrhythmias. TpTe and TpTe/QT were significantly increased in ST‐elevation myocardial infarction (STEMI) patients who experienced ventricular fibrillation (VF) within 24 hours of admission.^35^ Eslami et al. found that percutaneous coronary intervention (PCI) reduced both QTD and TpTe in patients presenting with STEMI.^36^ Interestingly, failure of these ECG parameters to improve after reperfusion was associated with development of major arrhythmias within 1 year.^37^ Post‐MI (myocardial infarction) patients who show clinical or inducible ventricular tachycardia (VT) have longer TpTe than those who are not inducible.^38^ TpTe is independently associated with all‐cause mortality as well as risk of fatal cardiac arrhythmia within the first year after ACS.^39^, ^40^

Repolarization heterogeneity is also linked to adverse long‐term outcomes in ACS populations. Haarmark et al. showed that pre‐PCI TpTe was associated with increased mortality in STEMI patients during 22 months of follow‐up.^41^ In 334 survivors of acute MI followed for 41 months, TCRT was an independent predictor of long‐term arrhythmic mortality.^42^ And, in patients who develop left ventricular systolic dysfunction post‐ACS, TpTe is independently predictive of implantable cardioverter defibrillator therapy and all‐cause mortality.^43^ Repolarization heterogeneity has also been linked to adverse outcomes in genetic arrhythmia syndromes, congenital heart disease, and valvular heart disease. In patients with Brudaga syndrome, TpTe and TpTe dispersion were prolonged in patients with recurrent aborted SCD or syncope compared to asymptomatic individuals.^44^ TpTe and TpTe/QT were predictive of VT/VF inducibility in Brugada patients undergoing programmed ventricular stimulation.^45^ Patients with repaired tetralogy of Fallot were found to have increased QTcD and TpTe compared to healthy controls.^46^ QTD was increased in patients with mitral valve prolapse and ventricular arrhythmias on Holter, compared to matched controls. QTD and QTcD were increased in patients with aortic stenosis, compared to controls, and were linearly related to disease severity.^47^ In patients with hypertrophic cardiomyopathy, QTD and markers of T‐wave complexity were increased, compared to controls, and were significantly greater in symptomatic patients.^48^, ^49^ TpTe has also been predictive of outcomes in other populations, including end‐stage renal disease, LV hypertrophy (LVH), and hypertension, as summarized in Table S1.^50^, ^51^, ^52^

## Mechanical Abnormalities in Patients With LQTS

Studies in LQTS have revealed that electrical repolarization abnormalities were accompanied by abnormal myocardial mechanics. Nador et al. noted that patients with LQTS had a more‐rapid early phase of ventricular contraction, as noted by a decreased time to early contraction (Th1/2). Hence, they reached half maximal systolic contraction more rapidly than controls. Furthermore, slow‐speed thickening in late systole, termed TsTh, was increased in LQTS patients, indicating that they spend more time at a low thickening rate. Taken together, rapid early contraction and prolonged slow thickening phase represent a particular pattern of abnormal myocardial mechanisms that was observed more frequently in symptomatic versus asymptomatic patients.^53^ The same group then assessed the response of these contraction abnormalities to verapamil. Verapamil was associated with increase in Th1/2 and reduction in TSTh, with normalization of the abnormal thickening patterns at peak effect. They suggested that symptomatic LQTS patients may have an abnormal increase in transcellular Ca levels which normalized with administration of Ca‐channel blockers.^54^

Haugaa et al. used tissue Doppler to show that contraction duration was longer in LQTS patients with past cardiac events compared to those without.^55^ Prolonged contraction duration showed higher specificity and sensitivity than QTc at predicting events. Greatest heterogeneity in contraction was observed in symptomatic LQTS mutation carriers compared to asymptomatic carriers or controls. The investigators concluded that prolonged myocardial contraction may lead to heterogeneous and delayed onset of the tissue Doppler e′ wave, implying diastolic dysfunction.^55^ Strain analysis confirmed longer mean contraction duration in symptomatic LQTS mutation carriers compared to asymptomatic carriers and healthy controls. Contraction duration by longitudinal strain was longer than by circumferential strain in symptomatic patients, suggesting increased transmural dispersion.^56^ Haguaa et al. recently demonstrated reduced global longitudinal strain in subjects with LQTS compared to healthy controls, as well as reduced e′ velocity (implying impaired LV relaxation) and increased left atrial volume index.^57^ Table S2 summarizes studies that have found associations between echocardiographic parameters and repolarization heterogeneity.

## Electromechanical Heterogeneity in Heart Failure

Early work by Alessandrini et al. revealed that electrocardiographic repolarization changes in T‐wave amplitude and QT interval induced through ventricular pacing were accompanied by echocardiographic changes in peak left ventricular filling rate and isovolumic relaxation time (IVRT). Specifically, QT prolongation was associated with increase IVRT and T‐wave amplitude was correlated with increase in peak LV filling rate. These findings were not accompanied by changes in systolic function and thus could not be explained on this basis. This link between electrical repolarization and diastolic mechanics may be, in part, mediated by the effects of calcium handling.^58^ Specifically, increase in APD (as was observed with ventricular pacing) has been linked to near doubling of cellular calcium influx and marked slowing of its decline.^59^ It is thus not surprising that prolonged APD may be associated with changes in LV relaxation and filling, processes that are calcium dependent. When relaxation and filling are most abnormal, in end‐stage heart failure, mapping of coronary‐perfused LV wedge preparations from human hearts confirmed prolongation of APD.^60^

**Figure 4:**
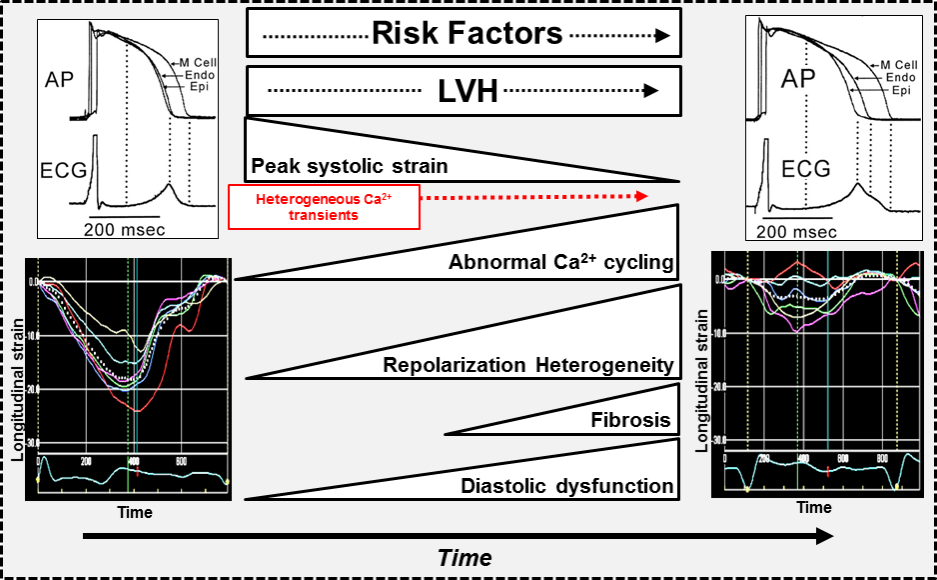
Proposed mechanism of electromechanical heterogeneity as a marker or contributor to heart failure. Progression from normal (left‐hand side) to overt heart failure (right‐hand side) is propagated by accumulation of risk factors such as hypertension, coronary artery disease, and diabetes and their consequences, which include left ventricular hypertrophy, heterogeneous dysregulation of Ca handling, and fibrosis. This manifests as electrical repolarization heterogeneity and abnormal myocardial mechanics, which includes diastolic dysfunction, reduction in peak longitudinal systolic strain, as well as contraction‐relaxation heterogeneity observed using strain imaging. AP indicates action potential; Endo, endocardial; Epi, epicardial; LVH, left ventricular hypertrophy; M Cell, Masonic myocardial Moe cell.

During the progression from structural heart disease to heart failure, there is development of an extensive amount of intercellular variability in Ca^2+^ kinetics. Disorganization in T tubules and impairment in Ca^2+^ cycling accompany reductions in absolute strain values and tissue Doppler velocities in spontaneously hypertensive rats.^61^ Initial myocardial remodeling is associated with heterogeneous increase in Ca^2+^ transient duration, which may, in part, explain the development of diastolic dysfunction as the result of a prolonging in relaxation. These changes in strain and diastolic function occur early in the remodeling process and precede the development of cardiac fibrosis and overt LV systolic dysfunction (Figure 4).^61^

Additionally, derangement in calcium cycling leads to increased vulnerability to intercellular repolarization gradients and cellular Ca^2+^ alternans, a setup for reentrant and triggered ventricular arrhythmias. Deficient sarcoplasmic reticulum Ca^2+^ uptake has been identified in cardiac myocytes from failing human hearts and has been linked to a decrease in expression and activity of the enzyme, Ca^2+^‐ATPase (SERCA2a). In animal models, transfection of SERCA2a reduced Ca^2+^ alternans, decreasing susceptibility to ventricular arrhythmias.^62^ In the Calcium Upregulation by Percutaneous Administration of Gene Therapy in Cardiac Disease (CUPID) gene therapy trial, intracoronary delivery of SERCA2a was associated with decreased events, clinical improvement in heart failure symptoms, as well as LV remodeling.^63^

**Figure 5:**
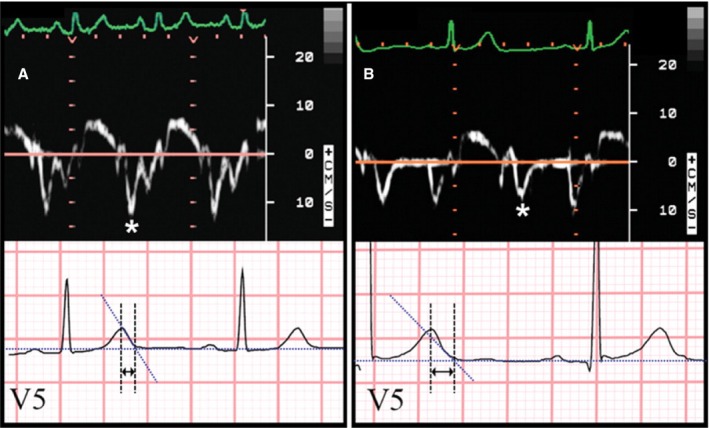
Correlation between tissue Doppler and ECG markers of repolarization heterogeneity. Shown here is an example of the relationship between tissue Doppler e′ velocity and ECG TpTe interval. A, Normal e′ velocity (12.1 cm/s) and short TpTe interval (65 ms). B, Abnormally reduced e′ velocity (7.8 cm/s) and long TpTe interval (115 ms). Asterisks denote e′ wave on tissue Doppler tracings. Arrows denote TpTe interval on ECG tracings. Reproduced from Sauer et al.^65^ Promotional and commercial use of the material in print, digital, or mobile device format is prohibited without the permission from the publisher Wolters Kluwer Health. ECG indicates electrocardiogram.

Finally, it has recently been shown that QTc correlates with severity of diastolic dysfunction on echocardiography in the general population, as well as in patients with clinical symptoms of heart failure.^64^ Sauer et al. recently showed that increased baseline TpTe was inversely associated with e′ velocity as well as peak exercise E/e′ ratio (Figure 5).^65^ Additionally, a linear association was noted between TpTe and SD in time to peak radial strain, a measure of heterogeneity in contraction duration. This finding was novel in establishing that the link between electrical repolarization and myocardial contractility occurred in patients without LQTS or significant cardiomyopathy.^66^ “Excitation‐contraction” coupling may represent a unifying theory linking the subclinical changes in myocardial dysfunction, calcium handling, and repolarization abnormalities with the development of symptomatic heart failure syndromes.

## Unanswered Questions

Cellular work, animal models, and human cohorts have all suggested that heterogeneity in myocardial repolarization exists, is increased in numerous disease states, and appears to confer increased cardiovascular risk. Unfortunately to date there is no consensus as to how best to measure repolarization heterogeneity in human subjects, given that various techniques in open‐chest animal models have revealed conflicting results. Furthermore, controversy also exists as to whether commonly used ECG measures, such as TpTe or QTD, truly represent the repolarization heterogeneity itself or are simply a surrogate for it. Future efforts should focus on prospectively assessing outcomes to determine which of the currently identified ECG measures of repolarization heterogeneity provides the greatest predictive value.

Furthermore, there is limited understanding currently as the role of repolarization heterogeneity in the development or progression of heart failure syndromes. Recent work revealed an association between abnormal TpTe duration and increased abnormalities in diastolic function, as well as several other structural and mechanical myocardial abnormalities. It is not yet clear whether increased repolarization heterogeneity is a marker of myocardial mechanical dysfunction, let alone causative. Understanding the role of repolarization heterogeneity in clinical heart failure symptoms represents a promising avenue of future investigation. Demonstrating independent association of repolarization heterogeneity with outcomes in heart failure populations would be noteworthy, given that it may identify a subgroup of patients that could uniquely be tailored for certain heart failure therapies. Similarly, whether normalization of repolarization heterogeneity with medical therapy is associated with improved outcomes in heart failure represents another avenue for further research. If improvements in ejection fraction are associated with decrease in repolarization heterogeneity, then perhaps identifying a pharmacological intervention to restore repolarization to a more‐normal state may have a role in improving cardiovascular risk for numerous populations.

## Conclusions

Accumulating evidence suggests that repolarization heterogeneity, a process initially understood at the cellular level through work on canine wedge preparations, may play a significant role in the pathophysiology of several cardiovascular conditions. Though initially linked to increased risk of arrhythmia in patients with inherited LQTSs, repolarization heterogeneity is now known to predict sudden death even in the general population. Analysis of T‐wave characteristics on the ECG may help in risk stratification in multiple cardiovascular conditions. More‐recent work linking repolarization heterogeneity to abnormalities in myocardial mechanics may provide insight into development and progression of clinical heart failure syndromes. Whether drugs that stabilize repolarization heterogeneity can improve electromechanical abnormalities or clinical outcomes requires further analysis. Additional studies are needed to identify other populations in which repolarization heterogeneity may confer risk, and determine whether targeting these electrical and mechanical abnormalities leads to improved cardiovascular outcomes.

## Sources of Funding

Shah is supported by grants from the National Institutes of Health (R01 HL10577 and R01 HL127028).

## Disclosures

None.

## Supporting information


**Table S1.** Studies of Clinical Outcomes Related to Increased Repolarization Heterogeneity
**Table S2.** Echocardiographic Parameters Associated With Repolarization Heterogeneity
