# Supplementary material for: Repolarization Heterogeneity: Beyond the QT Interval
Source: J Am Heart Assoc. 2016 Apr 29;5(5):e003607. doi: 10.1161/JAHA.116.003607 (PMC4889211; doi:10.1161/JAHA.116.003607)
Supplement: Supplementary file 1 — Table S1. Studies of Clinical Outcomes Related to Increased Repolarization Heterogeneity Table S2. Echocardiographic Parameters Associated With Repolarization Heterogeneity [file JAH3-5-e003607-s001.pdf]

SUPPLEMENTAL MATERIAL

Table S1. Studies of clinical outcomes related to increased repolarization heterogeneity

Authors	Population	Size	Metric	Results
Okin et al, 2002[1]	American Indians	1839	QTD and PCA	PCA ratio was an independent predictor of CV mortality in men and women. QTD was a significant predictor in women only
Okin et al, 2005[2]	American Indians	1729	PCA, TWR	Increased PCA ratio and TWR were significant predictors of CV mortality and TWR of all-cause mortality
Okin et al, 2004[3]	American Indians with DM	994	PCA	PCA ratio was an independent predictor of CV and all cause mortality
Ducceschi et al, 1998[4]	Aortic stenosis	70	QTD, QTcD	QTD and QTcD both linearly correlated with severity of aortic stenosis
Castro Hevia et al, 2006[5]	Brugada	58	TpTe	TpTe was prolonged in patients with recurrent VT/VF
Letsas et al, 2010[6]	Brugada	23	TpTe and TpTe/QT	TpTe and TpTe/QT were increased in patients with inducible VT/VF
Morin et al 2012[7]	Decreased EF	327	TpTe	TpTe predicted of ICD therapy, all-cause mortality, and the composite endpoint of ICD therapy or death
Lin et al, 2007[8]	ESRD	325	TWR	TWR was an independent predictor of CV and arrhythmia-related mortality
Tun et al, 1999[9]	ESRD	188	QTcD, TpTe	QTcD and TpTe were significantly higher in the ESRD group compared to controls
Shimizu et al, 2002[10]	HCM	47	TpTe/QT, QTD	QTD increased in HCM. TpTe/QT was increased in HCM patients with SCD/VT
Yetman et al, 1998[11]	HCM	99	QTcD	QTcD was associated with reduced time to death or resuscitated SCD

Yi et al, 1998[12]	HCM	83	QTD	QTD was increased in patients with symptomatic HCM and worse NYHA class
Kardys et al, 2003 [13]	Healthy	6134	QRST angle	Abnormal QRST angle was associated with cardiac death, non-fatal cardiac events, sudden death, and total mortality
Porthan et al, 2009[14]	Healthy	5917	PCA, TCRT, TWR	PCA ratio was independent predictor of all-cause and CV mortality in men. In women, independent mortality predictors were total TCRT (CV mortality) and TWR (all-cause and CV mortality)
Ferrucci et al, 2015[15]	HTN	40	TpTe	TpTe was higher in hypertensive than in normotensive individuals
Saba et al, 2005[16]	LVH	300	TpTe	TpTe increased in patients with LVH compared to controls
Smetana, et al 2011[17]	Male CV patients	813	TpTe, TWR	TpTe was shorter in non-survivors
Zabel et al, 2002[18]	Male CV patients	813	TWR	TWR was an independent predictor of all-cause mortality
Tieleman et al, 1995 [19]	MVP	64	QTD	QTD was increased in patients with MVP and ventricular arrhythmias
Watanabe et al, 2004[20]	NSVT, VT, VF, syncope, SVT	130	TpTe	TpTe was greatest in the VT inducible and VT spontaneous groups
de Bruyne MC, 1998[21]	Patients age >55	5812	QTD	Highest tertile relative to the lowest tertile had a twofold risk for cardiac death and sudden cardiac death, and 40% increased risk for total mortality
Batchvarov et al, 2004[22]	Post MI	334	TCRT	TCRT was an independent predictor of cardiac and arrhythmic mortality

Bonnemeier et al, 2001 [22]	Post MI	97	QTV	PCI was associated with decrease in QTV. Failure of QTV to decrease following reperfusion was associated with subsequent arrhythmic events
Erikssen et al, 2012[23]	Post MI	1359	TpTe	TpTe was a predictor of death and fatal cardiac arrhythmia
Eslami et al, 2013[24]	Post MI	80	TpTe, QTD	QTD and TpTe were reduced following PCI
Haarmark et al, 2009[25]	Post MI	101	TpTe	Pre-PCI TpTe interval predicted subsequent all-cause mortality
Lubinski et al, 2000[26]	Post MI	34	TpTe	TpTe increased in patients with inducible VT
Oikarinen et al, 2001 [27]	Post MI	73	TpTe	TpTe was increased in patients with inducible VT
Perkiomakl et al, 2006[28]	Post MI	437	TWLD, TCRT	TWLD was an independent predictor of cardiac mortality, TCRT was not
Shenthar et al, 2015[29]	Post MI	100	TpTe	TpTe and TpTe/QT were prolonged in patients post MI compared with healthy individuals, and predicted acute ventricular arrhythmias
Tatlisu et al, 2014[30]	Post MI	488	TpTe	TpTe interval was associated with in-hospital VT/VF, target vessel revascularization, death, as well as long-term target vessel revascularization and death
Zabel et al, 1998[31]	Post MI	280	TpTe, QTD	TpTe and QTD did not predict VT/VF or death in post MI patients
Zabel et al, 2000[32]	Post MI	280	TCRT, TWLD	TCRT but not TWLD, yielded independent predictive value of numerous CV outcomes
Savelieva et al, 1998[33]	Post MI, HCM	156	TpTe, QTD	TpTe was increased in HCM but not MI

				patients. QTD was significantly greater in both, compared to controls
Sarubbi et al, 1999[34]	Repaired TOF	74	QTD, TpTe	TOF patients have increase QTD and TpTe interval
Panikkath et al, 2011[35]	SCD	695	TpTe	TpTe was increased in patients with SCD than in controls. Odds of SCD increased stepwise with increase in TpTe
Pye et al, 1994[36]	VT	109	QTD	QTD increased in patients with sustained ventricular arrhythmias compared with controls

CV, cardiovascular; DM, diabetes mellitus; EF, ejection fraction; ESRD, end stage renal disease; HCM, hypertrophic cardiomyopathy; HTN, hypertension; ICD, implantable cardiac defibrillator; LVH, left ventricular hypertrophy; MI, myocardial infarction; MVP, mitral valve prolapse; NSVT, non sustained ventricular tachycardia; NYHA, New York Heart Association; PCA, principal component analysis; PCI percutaneous coronary intervention; QTcD, QTc dispersion; QTD, QT dispersion; QTV, QT variability; SCD, sudden cardiac death; SVT, supraventricular tachycardia; TCRT, total cosine R to T; TOF, tetralogy of fallot; TpTe, T peak T end; TWLD, T wave loop dispersion; TWR, T wave residuum; VF, ventricular fibrillation; VT, ventricular tachycardia

Table S2. Echocardiographic parameters associated with repolarization heterogeneity

Author	Parameter	Definition	Echo mode
Nador et al, 1991[37]	Thl/2	Time to reach half of maximal systolic thickening	MM
Nador et al, 1991[37]	TSTh	Slow movement in the late thickening phase	MM
Nakayama et al, 1998[38]	Wall thickening time (ThT)	Period in which the instantaneous wall thickness exceeds 90% of the maximum wall thickness	MM
Mayet et al, 1996[39]	E/A ratio	Ratio of early and late mitral inflow velocities	PWD
Sauer et al, 2012[40]	Diastolic dysfunction grade	Composite score using mitral inflow patters, TD velocity, and left atrial size	PWD, TD
Sauer et al, 2012[40]	E/E’	Ratio of early mitral valve inflow velocity to TD velocity	PWD, TD
Haugaa at al, 2010[41]	Delta contraction duration	Time difference between the longest and shortest contraction durations	Strain
Haugaa et al, 2010[41]	Mean contraction duration	Time from ECG onset of the R wave to maximum myocardial shortening	Strain
Haugaa et al, 2010[41]	Mechanical dispersion	Time difference in longitudinal and circumferential contraction duration in the 6 basal LV segments	Strain
Haugaa, et al 2009[42]	E'	Peak E' velocity	TD
Haugaa, et al 2009[42]	Post ejection velocity	Upstroke of the biphasic spike after	TD

		ejection.	
Haugaa, et al 2009[42]	Onset E' wave	Time from start of R wave to onset of E'	TD
Haugaa, et al 2009[42]	Contraction duration by velocity	Time from start of R wave to end of post ejection velocity	TD
Savoye et al, 2003[43]	Isovolumic relaxation time	Time between closure of the aortic valve and the opening of the mitral valve	TD
Savoye et al, 2003[43]	VHT	Deceleration time to reach half of maximal systolic velocity	TD
Savoye et al, 2003[43]	Peak systolic and diastolic velocity	Systolic and diastolic peak velocity	TD

MM, m-mode; TD, tissue doppler; PWD, pulsed wave doppler

Supplemental References

1. Okin PM, Devereux RB, Fabsitz RR, Lee ET, Galloway JM, Howard BV. Principal component analysis of the t wave and prediction of cardiovascular mortality in american indians: The strong heart study. *Circulation*. 2002;105:714-719
2. Okin PM, Malik M, Hnatkova K, Lee ET, Galloway JM, Best LG, Howard BV, Devereux RB. Repolarization abnormality for prediction of all-cause and cardiovascular mortality in american indians: The strong heart study. *J Cardiovasc Electrophysiol*. 2005;16:945-951
3. Okin PM, Devereux RB, Lee ET, Galloway JM, Howard BV, Strong Heart S. Electrocardiographic repolarization complexity and abnormality predict all-cause and cardiovascular mortality in diabetes: The strong heart study. *Diabetes*. 2004;53:434-440
4. Ducceschi V, Sarubbi B, D'Andrea A, Liccardo B, Briglia N, Carozza A, Marmo J, Santangelo L, Iacono A, Cotrufo M. Increased qt dispersion and other repolarization abnormalities as a possible cause of electrical instability in isolated aortic stenosis. *Int J Cardiol*. 1998;64:57-62
5. Castro Hevia J, Antzelevitch C, Tornes Barzaga F, Dorantes Sanchez M, Dorticos Balea F, Zayas Molina R, Quinones Perez MA, Fayad Rodriguez Y. Tpeak-tend and tpeak-tend dispersion as risk factors for ventricular tachycardia/ventricular fibrillation in patients with the brugada syndrome. *J Am Coll Cardiol*. 2006;47:1828-1834
6. Letsas KP, Weber R, Astheimer K, Kalusche D, Arentz T. Tpeak-tend interval and tpeak-tend/qt ratio as markers of ventricular tachycardia inducibility in subjects with brugada ecg phenotype. *Europace*. 2010;12:271-274
7. Morin DP, Saad MN, Shams OF, Owen JS, Xue JQ, Abi-Samra FM, Khatib S, Nelson-Twakor OS, Milani RV. Relationships between the t-peak to t-end interval, ventricular tachyarrhythmia, and death in left ventricular systolic dysfunction. *Europace*. 2012;14:1172-1179
8. Lin CY, Lin LY, Chen PC. Analysis of t-wave morphology from the 12-lead electrocardiogram for prediction of long-term prognosis in patients initiating haemodialysis. *Nephrol Dial Transplant*. 2007;22:2645-2652
9. Tun A, Khan IA, Wattanasauwan N, Win MT, Hussain A, Hla TA, Cherukuri VL, Vasavada BC, Sacchi TJ. Increased regional and transmyocardial dispersion of ventricular repolarization in end-stage renal disease. *Can J Cardiol*. 1999;15:53-56
10. Shimizu M, Ino H, Okeie K, Yamaguchi M, Nagata M, Hayashi K, Itoh H, Iwaki T, Oe K, Konno T, Mabuchi H. T-peak to t-end interval may be a better predictor of high-risk patients with hypertrophic cardiomyopathy associated with a cardiac troponin i mutation than qt dispersion. *Clin Cardiol*. 2002;25:335-339
11. Yetman AT, Hamilton RM, Benson LN, McCrindle BW. Long-term outcome and prognostic determinants in children with hypertrophic cardiomyopathy. *J Am Coll Cardiol*. 1998;32:1943-1950
12. Yi G, Elliott P, McKenna WJ, Prasad K, Sharma S, Guo XH, Camm AJ, Malik M. Qt dispersion and risk factors for sudden cardiac death in patients with hypertrophic cardiomyopathy. *Am J Cardiol*. 1998;82:1514-1519
13. Kardys I, Kors JA, van der Meer IM, Hofman A, van der Kuip DA, Witteman JC. Spatial qrs-t angle predicts cardiac death in a general population. *Eur Heart J*. 2003;24:1357-1364

14. Porthan K, Viitasalo M, Jula A, Reunanen A, Rapola J, Vaananen H, Nieminen MS, Toivonen L, Salomaa V, Oikarinen L. Predictive value of electrocardiographic qt interval and t-wave morphology parameters for all-cause and cardiovascular mortality in a general population sample. *Heart Rhythm*. 2009;6:1202-1208, 1208 e1201
15. Ferrucci A, Canichella F, Battistoni A, Palano F, Francia P, Ciavarella GM, Volpe M, Tocci G. A novel electrocardiographic t-wave measurement (tp-te interval) as a predictor of heart abnormalities in hypertension: A new opportunity for first-line electrocardiographic evaluation. *J Clin Hypertens (Greenwich)*. 2015; 17:441-9
16. Saba MM, Arain SA, Lavie CJ, Abi-Samra FM, Ibrahim SS, Ventura HO, Milani RV. Relation between left ventricular geometry and transmural dispersion of repolarization. *Am J Cardiol*. 2005;96:952-955
17. Smetana P, Schmidt A, Zabel M, Hnatkova K, Franz M, Huber K, Malik M. Assessment of repolarization heterogeneity for prediction of mortality in cardiovascular disease: Peak to the end of the t wave interval and nondipolar repolarization components. *J Electrocardiol*. 2011;44:301-308
18. Zabel M, Malik M, Hnatkova K, Papademetriou V, Pittaras A, Fletcher RD, Franz MR. Analysis of t-wave morphology from the 12-lead electrocardiogram for prediction of long-term prognosis in male us veterans. *Circulation*. 2002;105:1066-1070
19. Tieleman RG, Crijns HJ, Wiesfeld AC, Posma J, Hamer HP, Lie KI. Increased dispersion of refractoriness in the absence of qt prolongation in patients with mitral valve prolapse and ventricular arrhythmias. *Br Heart J*. 1995;73:37-40
20. Watanabe N, Kobayashi Y, Tanno K, Miyoshi F, Asano T, Kawamura M, Mikami Y, Adachi T, Ryu S, Miyata A, Katagiri T. Transmural dispersion of repolarization and ventricular tachyarrhythmias. *Journal of Electrocardiology*. 2004;37:191-200
21. de Bruyne MC, Hoes AW, Kors JA, Hofman A, van Bemmel JH, Grobbee DE. Qtc dispersion predicts cardiac mortality in the elderly: The rotterdam study. *Circulation*. 1998;97:467-472
22. Batchvarov VN, Hnatkova K, Poloniecki J, Camm AJ, Malik M. Prognostic value of heterogeneity of ventricular repolarization in survivors of acute myocardial infarction. *Clin Cardiol*. 2004;27:653-659
23. Erikssen G, Liestol K, Gullestad L, Haugaa KH, Bendz B, Amlie JP. The terminal part of the qt interval (t peak to t end): A predictor of mortality after acute myocardial infarction. *Ann Noninvasive Electrocardiol*. 2012;17:85-94
24. Eslami V, Safi M, Taherkhani M, Adibi A, Movahed MR. Evaluation of qt, qt dispersion, and t-wave peak to end time changes after primary percutaneous coronary intervention in patients presenting with acute st-elevation myocardial infarction. *J Invasive Cardiol*. 2013;25:232-234
25. Haarmark C, Hansen PR, Vedel-Larsen E, Pedersen SH, Graff C, Andersen MP, Toft E, Wang F, Struijk JJ, Kanters JK. The prognostic value of the tpeak-tend interval in patients undergoing primary percutaneous coronary intervention for st-segment elevation myocardial infarction. *J Electrocardiol*. 2009;42:555-560
26. Lubinski A, Kornacewicz-Jach Z, Wnuk-Wojnar AM, Adamus J, Kempa M, Krolak T, Lewicka-Nowak E, Radomski M, Swiatecka G. The terminal portion of the t wave: A new electrocardiographic marker of risk of ventricular arrhythmias. *Pacing Clin Electrophysiol*. 2000;23:1957-1959
27. Oikarinen L, Viitasalo M, Korhonen P, Vaananen H, Hanninen H, Montonen J, Makijarvi M, Katila T, Toivonen L. Postmyocardial infarction patients susceptible to ventricular tachycardia show increased t wave dispersion independent of delayed ventricular conduction. *J Cardiovasc Electrophysiol*. 2001;12:1115-1120

28. Perkiomaki JS, Hyytinen-Oinas M, Karsikas M, Seppanen T, Hnatkova K, Malik M, Huikuri HV. Usefulness of t-wave loop and qrs complex loop to predict mortality after acute myocardial infarction. *Am J Cardiol*. 2006;97:353-360
29. Shenthar J, Deora S, Rai M, Nanjappa Manjunath C. Prolonged tpeak-end and tpeak-end/qt ratio as predictors of malignant ventricular arrhythmias in the acute phase of st-segment elevation myocardial infarction: A prospective case-control study. *Heart Rhythm*. 2015;12:484-489
30. Tatlisu MA, Ozcan KS, Gungor B, Ekmekci A, Cekirdekci EI, Arugarslan E, Cinar T, Zengin A, Karaca M, Eren M, Erdinler I. Can the t-peak to t-end interval be a predictor of mortality in patients with st-elevation myocardial infarction? *Coron Artery Dis*. 2014;25:399-404
31. Zabel M, Klingenheben T, Franz MR, Hohnloser SH. Assessment of qt dispersion for prediction of mortality or arrhythmic events after myocardial infarction: Results of a prospective, long-term follow-up study. *Circulation*. 1998;97:2543-2550
32. Zabel M, Acar B, Klingenheben T, Franz MR, Hohnloser SH, Malik M. Analysis of 12-lead t-wave morphology for risk stratification after myocardial infarction. *Circulation*. 2000;102:1252-1257
33. Savelieva I, Yap YG, Yi G, Guo X, Camm AJ, Malik M. Comparative reproducibility of qt, qt peak, and t peak-t end intervals and dispersion in normal subjects, patients with myocardial infarction, and patients with hypertrophic cardiomyopathy. *Pacing Clin Electrophysiol*. 1998;21:2376-2381
34. Sarubbi B, Pacileo G, Ducceschi V, Russo MG, Iacono C, Pisacane C, Iacono A, Calabro R. Arrhythmogenic substrate in young patients with repaired tetralogy of fallot: Role of an abnormal ventricular repolarization. *Int J Cardiol*. 1999;72:73-82
35. Panikkath R, Reinier K, Uy-Evanado A, Teodorescu C, Hattenhauer J, Mariani R, Gunson K, Jui J, Chugh SS. Prolonged tpeak-to-tend interval on the resting ecg is associated with increased risk of sudden cardiac death. *Circ Arrhythm Electrophysiol*. 2011;4:441-447
36. Pye M, Quinn AC, Cobbe SM. Qt interval dispersion: A non-invasive marker of susceptibility to arrhythmia in patients with sustained ventricular arrhythmias? *Br Heart J*. 1994;71:511-514
37. Nador F, Beria G, De Ferrari GM, Stramba-Badiale M, Locati EH, Lotto A, Schwartz PJ. Unsuspected echocardiographic abnormality in the long qt syndrome. Diagnostic, prognostic, and pathogenetic implications. *Circulation*. 1991;84:1530-1542
38. Nakayama K, Yamanari H, Otsuka F, Fukushima K, Saito H, Fujimoto Y, Emori T, Matsubara H, Uchida S, Ohe T. Dispersion of regional wall motion abnormality in patients with long qt syndrome. *Heart*. 1998;80:245-250
39. Mayet J, Shahi M, McGrath K, Poulter NR, Sever PS, Foale RA, Thom SA. Left ventricular hypertrophy and qt dispersion in hypertension. *Hypertension*. 1996;28:791-796
40. Sauer A, Wilcox JE, Andrei AC, Passman R, Goldberger JJ, Shah SJ. Diastolic electromechanical coupling: Association of the ecg t-peak to t-end interval with echocardiographic markers of diastolic dysfunction. *Circ Arrhythm Electrophysiol*. 2012;5:537-543
41. Haugaa KH, Amlie JP, Berge KE, Leren TP, Smiseth OA, Edvardsen T. Transmural differences in myocardial contraction in long-qt syndrome: Mechanical consequences of ion channel dysfunction. *Circulation*. 2010;122:1355-1363
42. Haugaa KH, Edvardsen T, Leren TP, Gran JM, Smiseth OA, Amlie JP. Left ventricular mechanical dispersion by tissue doppler imaging: A novel approach for identifying high-risk individuals with long qt syndrome. *Eur Heart J*. 2009;30:330-337
43. Savoye C. Tissue doppler echocardiography in patients with long qt syndrome. *European Journal of Echocardiography*. 2003;4:209-213
